# Plasma Trimethylamine-N-Oxide Is an Independent Predictor of Long-Term Cardiovascular Mortality in Patients Undergoing Percutaneous Coronary Intervention for Acute Coronary Syndrome

**DOI:** 10.3389/fcvm.2021.728724

**Published:** 2021-10-29

**Authors:** Ceren Eyileten, Joanna Jarosz-Popek, Daniel Jakubik, Aleksandra Gasecka, Marta Wolska, Marcin Ufnal, Marek Postula, Aurel Toma, Irene M. Lang, Jolanta M. Siller-Matula

**Affiliations:** ^1^Department of Experimental and Clinical Pharmacology, Centre for Preclinical Research and Technology (CEPT), Medical University of Warsaw, Warsaw, Poland; ^2^First Chair and Department of Cardiology, Medical University of Warsaw, Warsaw, Poland; ^3^Department of Experimental Physiology and Pathophysiology, Laboratory of Centre for Preclinical Research, Medical University of Warsaw, Warsaw, Poland; ^4^Department of Internal Medicine II, Division of Cardiology, Medical University of Vienna, Vienna, Austria

**Keywords:** trimethylamine N-oxide, acute coronary syndrome, type 2 diabetes mellitus, cardiovascular mortality, cardiovascular disease

## Abstract

To investigate the association of liver metabolite trimethylamine N-oxide (TMAO) with cardiovascular disease (CV)-related and all-cause mortality in patients with acute coronary syndrome (ACS) who underwent percutaneous coronary intervention. Our prospective observational study enrolled 292 patients with ACS. Plasma concentrations of TMAO were measured during the hospitalization for ACS. Observation period lasted seven yr in median. Adjusted Cox-regression analysis was used for prediction of mortality. ROC curve analysis revealed that increasing concentrations of TMAO levels assessed at the time point of ACS significantly predicted the risk of CV mortality (c-index=0.78, *p* < 0.001). The cut-off value of >4 μmol/L, labeled as high TMAO level (23% of study population), provided the greatest sum of sensitivity (85%) and specificity (80%) for the prediction of CV mortality and was associated with a positive predictive value of 16% and a negative predictive value of 99%. A multivariate Cox regression model revealed that high TMAO level was a strong and independent predictor of CV death (HR = 11.62, 95% CI: 2.26–59.67; *p* = 0.003). High TMAO levels as compared with low TMAO levels were associated with the highest risk of CV death in a subpopulation of patients with diabetes mellitus (27.3 vs. 2.6%; *p* = 0.004). Although increasing TMAO levels were also significantly associated with all-cause mortality, their estimates for diagnostic accuracy were low. High TMAO level is a strong and independent predictor of long-term CV mortality among patients presenting with ACS.

## Introduction

Acute coronary syndrome (ACS) remains a leading cause of mortality worldwide (1). Despite development of pharmacological treatment and percutaneous coronary intervention (PCI) (2–5), patients who experienced ACS are at high risk of future cardiovascular events and death (6–8). Identification of reliable predictive tools could potentially improve the risk stratification (9). Numerous studies revealed that intestinal microbial organisms (microbiota) and its metabolites, as TMA (oxidize trimethylamine) may play a pathogenic role in a wide range of diseases, including the onset and progression of cardiovascular disease (CVD) and ACS (10). By oxidation of TMA in the liver originates TMAO (trimethylamine-N-oxide). An additional, direct source of TMAO in humans is TMAO-rich seafood (11). Elevated concentration of circulating TMAO has been associated with increased risk of CVD and major adverse cardiac events (MACE), including myocardial infarction (MI), stroke, major bleeding and all-cause mortality (12). Though, data on the effect of TMAO on the circulatory system are conflicting (11, 13–15).

A population of special interest in the context of bacterial metabolites are patients with diabetes mellitus (DM) (16, 17). Patients with DM are at a higher risk of developing micro- and macrovascular complications such as chronic kidney disease (CKD) or atherosclerosis than those without these risk factors (16, 17). Because the number of patients with DM continues to increase, there is a need for new CV risk prediction markers in order to reduce DM-related complications (18, 19). Elevated intake of TMAO precursors in the diet shows an association with a higher risk of developing DM (20). Moreover, recent studies revealed that DM contributes to even a 10-fold increase in TMAO levels, indicating a potential role of this metabolite as a predictor of insulin resistance and impaired glucose tolerance (21). It has been shown that the all-cause and CV-related mortality among DM individuals is up to 4 - fold higher compared to patients without DM (14).

Herein, our objective was to investigate the association of TMAO with CV-related and all-cause mortality in patients with ACS undergoing percutaneous coronary intervention (PCI) with a special focus on patients with DM.

## Methods

This is a prospective observational study, which included consecutive ACS patients and blood sampling was done between July 2012 until July 2016 at the Medical University of Vienna. The Ethics Committee of the Medical University of Vienna approved the study protocol in accordance with the Declaration of Helsinki. In summary, the study aimed to investigate short-term and long-term clinical outcomes in consecutive patients after ACS and PCI who were treated with potent platelet inhibitors as ticagrelor and prasugrel. Inclusion criteria were comprised ACS at admission, provision of written informed consent before study entry, age >18 yr and planned treatment with potent platelet inhibitors. Patients were excluded when participating in interventional trials. Follow-up information was obtained by contacting patients every three months within the first year. Further, data regarding long-term mortality was acquired through queries of the Austrian death registry - most recently, in June 2020. For this analysis we only considered patients, where blood biomarkers were assessed during hospitalization for ACS (22–24).

Primary efficacy endpoint was long-term CV death. Incidence of MACE within one year after discharge and long-term all-cause mortality were regarded as our secondary endpoint. MACE was determined as non-fatal MI, non-fatal stroke and CV death. The composite endpoint was defined in accordance with the current universal criteria (25, 26). Stroke was defined as an abrupt onset of a focal neurologic deficit, generally distributed in the territory of a single brain artery (including the retinal artery), and that is not attributable to an identifiable non-vascular cause (i.e., brain tumor or trauma). Stroke definition reflects the Statement for Healthcare Professionals From the American Heart Association/American Stroke Association (27), that incorporates the World Health Organization (WHO) definition of stroke (28). Myocardial infarction (MI) was defined according to the latest version of the Universal Definition (29). CV deaths include deaths that result from an acute myocardial infarction, sudden cardiac death, death due to heart failure, death due to stroke, death due to CV procedures, death due to CV hemorrhage, and death due to other CV causes according (26).

Venous blood samples were drawn (VACUETTE^®^ CAT Serum Sep Clot Activator; 8 mL). The evaluation of blood plasma metabolites concentration was done in December 2019 at the Medical University of Warsaw. The plasma concentration of TMAO was evaluated using a Waters Acquity Ultra Performance Liquid Chromatography coupled with a Waters TQ-S Triple-Quadrupole Mass Spectrometer as we have previously described (30, 31). In short, chromatographic separation was performed using a Waters HILIC column (1.7 μm, 2.1 mm × 50 mm) thermostatted at 70°C. Mobile phase A was Mili-Q water with an addition of 1 mL of 25% NH4OH per 1,000 mL of water, and mobile phase B was 1 mL of formic acid in 1,000 mL of acetonitrile. The flow rate of the mobile phase was set at 0.5 mL/min. The total time of separation was 1.7 min. The mass spectrometer operated in multiple-reaction monitoring (MRM)-positive electrospray ionization (ESI+) mode. The calibration curve ranges were 0.02–20 μg/mL for TMAO. Mean R2 coefficients of a calibration curves from 6 calibrators was not lower than 0.99.

Risk factors, clinical data and categorical variables are presented as percentages of patients and were compared using χ2 or Fisher's exact tests, as appropriate. Continuous data are expressed as mean ± standard deviation (SD) or median and interquartile range depending on the data distribution and compared using Student's *t* test, Mann-Whitney *U* test for two independent samples. The distribution of data was checked with the Kolmogorov-Smirnov test. The Kaplan-Meier method was utilized for construction of survival curves. The log-rank test was applied to evaluate differences between groups. Proportional Cox-regression analysis was used to adjust for confounding factors. Potential confounders (prior MI, diabetes, age, gender, diabetes, hemoglobin and creatinine levels at admission) were entered into the Cox model on the basis of known clinical relevance or significant association observed at univariate analysis. Effect estimates were presented as hazard ratios (HR) and 95% CI. All tests were two-sided, a *p*-value < 0.05 was considered statistically significant. Calculations were performed using SPSS version 22.0 (IBM Corporation, Chicago, USA). Based on a 16% long-term mortality in the high TMAO group as compared to 1% in the low TMAO group, we calculated that with 290 patients (3:1 sampling ratio), our analysis had 99% power with a two-sided alpha value of < 0.05.

## Results

**Figure 1:**
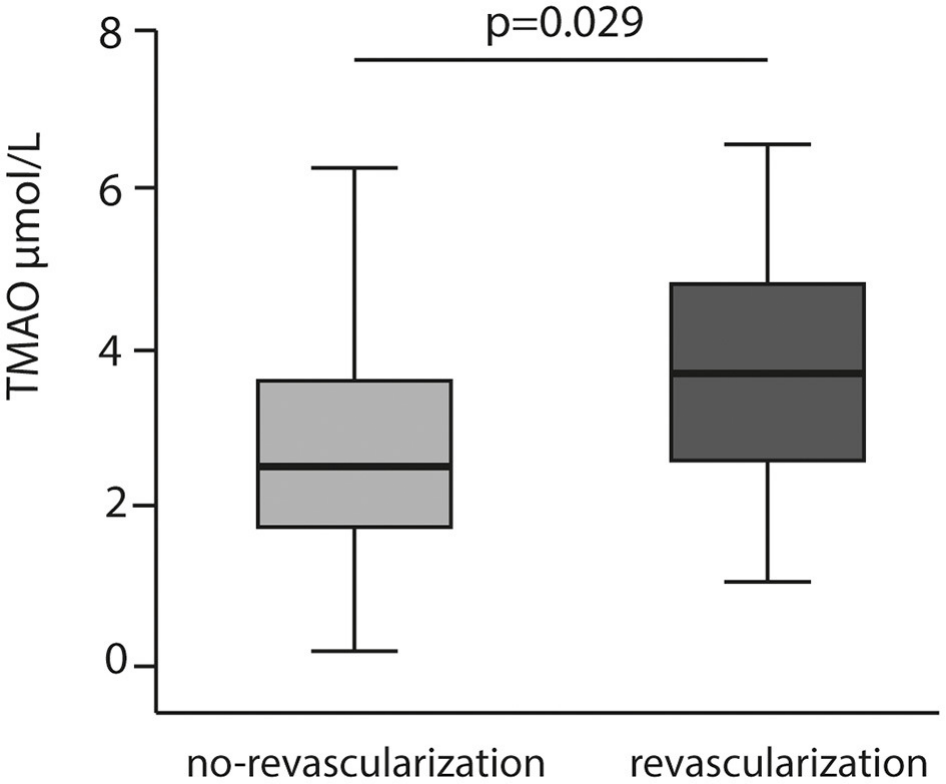
TMAO levels relation to repeated revascularization.

**Figure 2:**
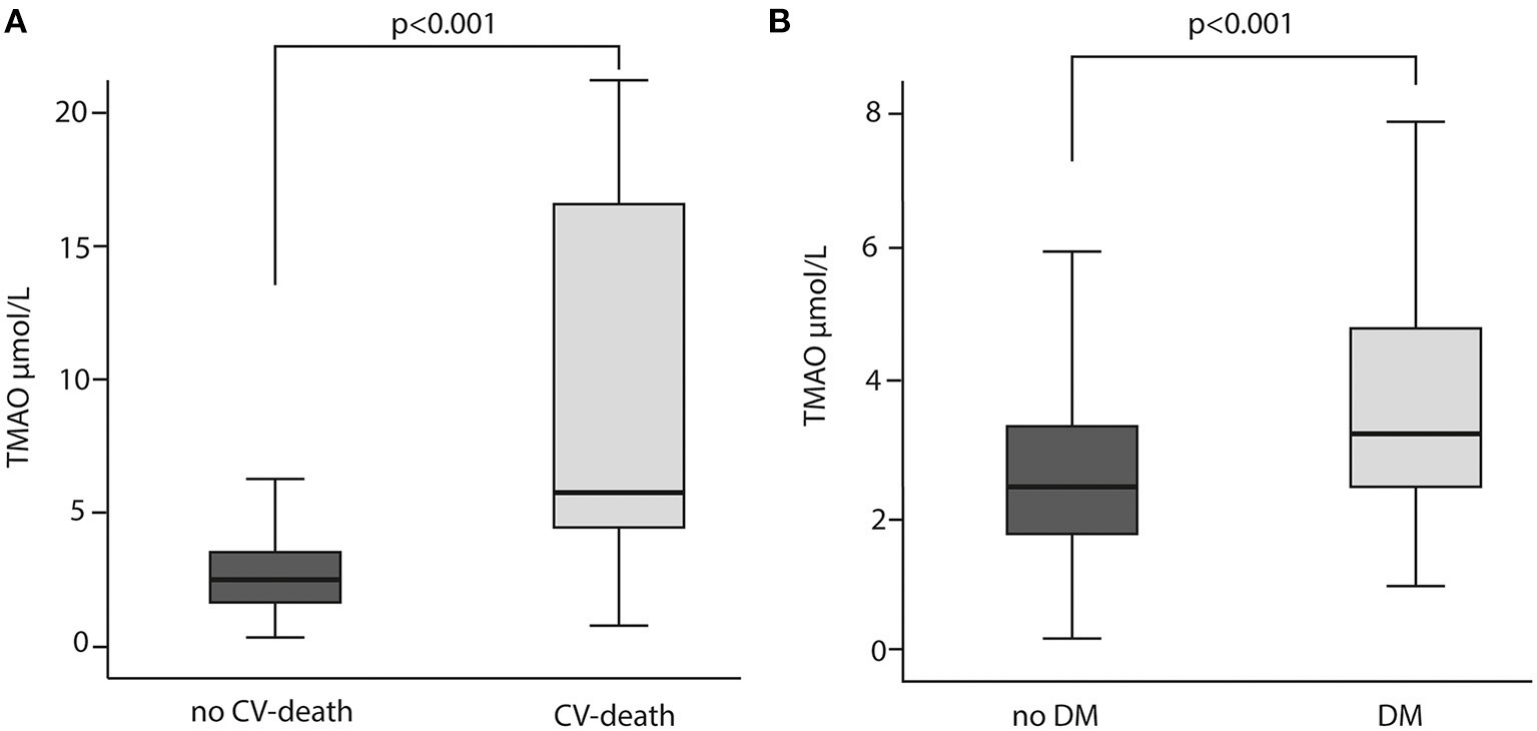
TMAO levels in relation to cardiovascular (CV) death **(A)** CV death and diabetes mellitus (DM) **(B)** Mann-Whitney *U* test was used for the comparison.

Patient demographics, concomitant medication, laboratory results and ACS data are summarized in Table 1. Overall, the majority of the participants were male (80%). Out of 344 patients included, blood samples for analyses presented in this study were unavailable for 52 subjects. Among the 292 patients included, 13 (5%) patients died within a median observation time of 84 months (7 years). The most frequent diagnosis at admission has been ST Elevation Myocardial Infarction (STEMI) with 63 % in total. CV risk factors, such as hypertension (63%), dyslipidemia (56%) and history of smoking (74%) were common in most patients. DM has been diagnosed in 20% of the cohort. Peripheral artery disease (PAD) and cerebrovascular disease were reported in 6 and 4%, respectively. Acetylsalicylic acid (ASA) was administered to 100% of the study population. Patients who underwent repeated revascularization (non-target or target vessel) had significantly higher TMAO concentration than patients who did not need revascularization (*p* = 0.029) (Figure 1). Patients who died had 3- fold higher TMAO levels as compared with patients who survived (*p* < 0.001; Figure 2A). Also, patients with DM had 1.4- fold higher TMAO levels than patients without DM (*p* < 0.001; Figure 2B).

**Table 1:** Patient demographics.

**Patient demographics**	**Overall** ***N*** **= 292 (100)**	**Low TMAO** ***N*** **= 225 (77)**	**High TMAO** ***N*** **= 67 (23)**	***p*** **-value**
TMAO (μmol/L) median and IQR	2.57 [1.77–3.85]	2.2 [1.55–2.84]	6.16 [4.84–8.13]	**<0.001**
Age (years)	59 ± 12	56 ± 11	66 ± 14	**<0.001**
Sex (male) *n* (%)	232 (80)	183 (81)	49 (73)	0.145
**Risk factors/past medical history** ***n*** **(%)**				
Body mass index (BMI)	27.8 ± 5.2	27.6 ± 5	28.4 ± 5	0.465
Hypertension	184 (63)	140 (63)	44 (66)	0.667
Dyslipidaemia	165 (57)	129 (58)	36 (54)	0.576
Diabetes mellitus	61 (21)	39 (17)	22 (33)	**0.006**
Peripheral artery disease	15 (5)	7 (3.1)	8 (12)	**0.004**
Cerebrovascular disease	11 (4)	8 (4)	3 (5)	0.733
Chronic obstructive pulmonary disease (COPD)	12 (4)	8 (4)	4 (6)	0.382
Smoking	215 (74)	173 (77)	42 (63)	**0.017**
Family history of CAD	146 (50)	118 (53)	27 (40.3)	0.075
Prior myocardial infarction	64 (22)	43 (19)	21 (31)	**0.035**
Prior PCI	32 (11)	20 (9)	12 (18)	**0.039**
**Laboratory data**				
White blood cell count (× 10^9^/L)	11 ± 4	11 ± 4	10.4 ± 4	0.159
Platelets (× 10^9^/L)	241.6 ± 69	246.4 ± 67	225 ± 75	**0.011**
Hemoglobin (g/dL)	14.6 ± 2	14.6 ± 1.6	13.9 ± 2.5	**0.011**
High-sensitivity C-reactive Protein (mg/dL)	1.14 [0.54–3.04]	1.16 [0.53–2.98]	1.02 [0.59–3.12]	0.825
Fibrinogen (mg/dL)	390.7 ± 97.5	386.3 ± 94.6	406.5 ± 107	0.300
Creatinine (mg/dL)	0.91 [0.8–1.07]	0.9 [0.79–1.01]	0.99 [0.86–1.24]	**<0.001**
Troponin T (μg/L)	0.08 [0.03–0.38]	0.08 [0.02–0.39]	0.08[0.04–33]	0.558
**Concomitant medications** ***n*** **(%)**				
ASA	292	225 (100)	67 (100)	
Ticagrelor	143 (95)	111 (49)	32 (48)	0.821
Prasugrel	149 (51)	114 (50)	35 (52)	0.821
ß-blockers (BB)	266 (93)	210 (96)	56 (86)	**0.005**
Angiotensin converting enzyme (ACE) inhibitors	251 (88)	192 (94)	59 (91)	0.494
Statins	265 (93)	202 (92)	63 (97)	0.184
Antidiabetic drugs	40 (14)	25 (12)	15 (23)	**0.018**
Calcium channel-blockers (CCB)	25 (9)	15 (7)	10 (15)	**0.033**
Proton pump Inhibitors (PPI)	214 (75)	167 (76)	47 (72)	0.517
**ACS data**				
Unstable Angina Pectoris	12 (4)	7 (3)	5 (7.5)	0.516
NSTEMI	106 (36)	81 (36)	25 (37)	0.823
STEMI	174 (60)	137 (61)	37 (55)	0.407
Number of stents per patient	1.4 ± 0.9	1.4 ± 1	1.5 ± 1	0.759
Total stent length	27.5 [18–38]	26.5 [18–38]	28 [17–44]	0.512

*Data are reported as mean ± standard deviation (SD), median and IQR depending on the distribution of the data and n (number of patients) or percentages; Student's t test, Mann-Whitney U test or Chi Square test used for the comparison. P value < 0.05 is presented bold. CAD, coronary artery disease; PCI, percutaneous coronary intervention, STEMI, ST-elevation myocardial infarction; NSTEMI, non-ST-elevation myocardial infarction*.

**Figure 3:**
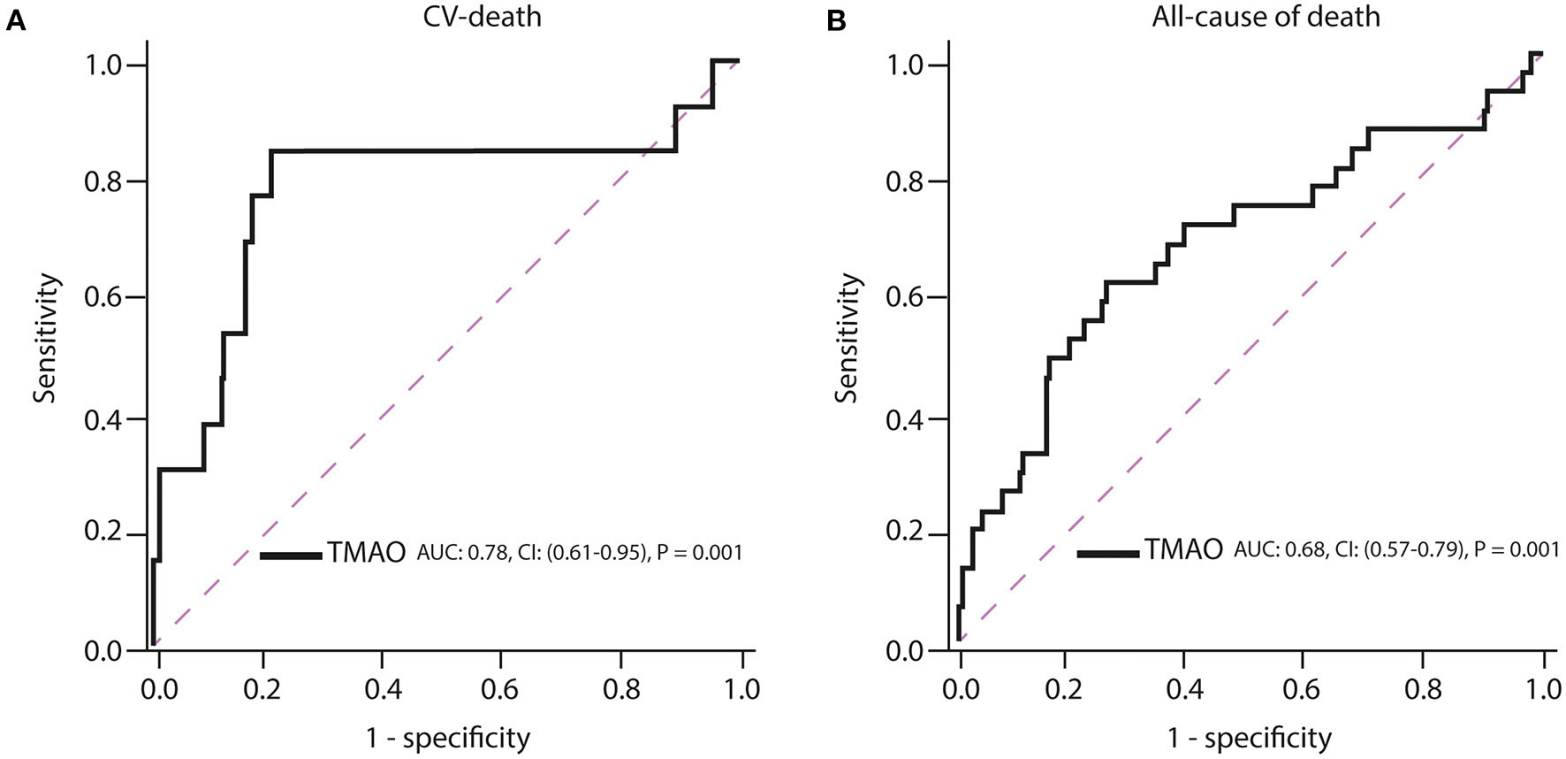
Receiver operating characteristic (ROC) curves of TMAO for prediction of **(A)** cardiovascular death and **(B)** all-cause of death. AUC, area under the curve; CI, confidence interval.

ROC curve analysis has shown that TMAO cut-off of >4 μmol/L (high TMAO group) had a c-index of 0.78 (95% (CI): 0.61-0.95; *p* = 0.001; Figure 2A, Table 2) for prediction of long-term CV death, which was associated with a 80% sensitivity, 85% specificity, 99% positive predictive value, 16% negative predictive value, and 4.2 positive likelihood ratio (Figure 3A, Table 2).

**Table 2:** Statistical estimates for prediction of cardiovascular death by metabolites.

**Test**	**c-index** **(95% CI)**	***P***	**Cut-off**	**Sensitivity,** **%**	**Specificity, %**	**Positive** **predictive** **value, %**	**Negative** **predictive** **value, %**	**Positive** **likelihood** **ratio**
TMAO (μmol/L)	0.78 (0.61–0.95)	0.001	4 μmol/L	85	80	16	99	4.3

Increasing TMAO levels were significantly associated with all-cause mortality, but their c-index estimates were low (TMAO: c-index = 0.68; 95% (CI): 0.57–0.79; *p* = 0.001; Figure 3B). The measures for diagnostic accuracy of TMAO for all-cause mortality were lower than for CV mortality (Tables 2, 3).

**Table 3:** Statistical estimates for prediction of all-cause of death by TMAO.

**Test**	**c-index- AUC** **(95% CI)**	***P***	**Cut-off**	**Sensitivity,** **%**	**Specificity, %**	**Positive** **predictive** **value, %**	**Negative** **predictive** **value, %**	**Positive** **likelihood** **ratio**
TMAO (μmol/L)	0.68 (0.57–0.79)	0.001	3.5 μmol/L	61	75	22	94	2.4

Based on the best TMAO accuracy cut-off, the study population (292 patients) was divided into two subgroups: low TMAO (mean ± standard deviation [SD]: 2.2 ± 0.85 μmol/L), including 225 (77%) patients and high TMAO (8.98 ± 11.46 μmol/L), counting 67 (23%) patients. Mean age in patients assigned to the low TMAO subgroup was 56 ± 11 yr and 66 ± 14 years in patients of the high TMAO subgroup (*p* < 0.0001). Likewise, DM (17 vs. 33%; *p* = 0.006), PAD (3 vs. 12%; *p* = 0.009), prior MI (19 vs. 31%; *p* = 0.039), prior PCI (9 vs. 18%; *p* = 0.035) were more frequent in patients with high TMAO than with low TMAO. Interestingly, smokers had more often low TMAO than high TMAO levels (77 vs. 63%; *p* = 0.017). Moreover, beta-blockers (BB) were applied more often in the low TMAO subgroup rather than in the high TMAO subgroup (96 vs. 86%; *p* = 0.009). Contrarily, patients with high TMAO levels were administered more frequent calcium channel-blockers (CCB) (15 vs. 7%; *p* = 0.033), and antidiabetic drugs (23 vs. 12%; *p* = 0.018). Regarding laboratory data, platelets and hemoglobin levels were higher in patients with low TMAO than high TMAO (246.4 ± 67 × 10^9^/L vs. 225 ± 75 × 10^9^/L; *p* = 0.011 and 14.6 ± 1.6 g/dL vs. 13.9 ± 2.5 g/dL; *p* = 0.011, respectively) concentrations. Creatinine values were higher in patients with high TMAO levels (1.1 ± 0.6 vs. 0.9 ± 1.2; *p* < 0.001).

**Figure 4:**
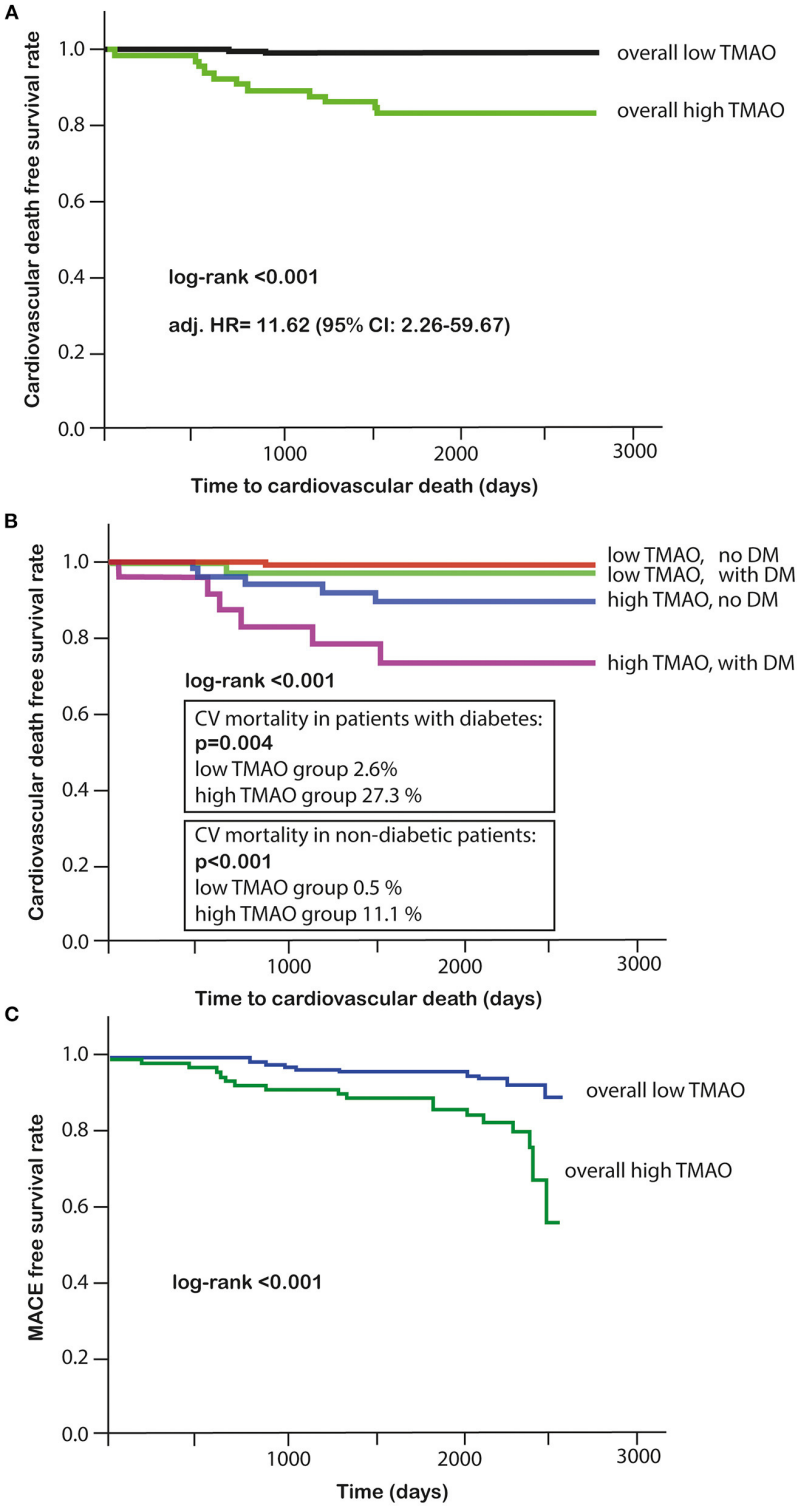
**(A)** Kaplan-Meier survival analysis for cardiovascular death in high or low TMAO subgroups; **(B)** Kaplan-Meier survival analysis in DM and high or low TMAO subgroups; **(C)** Kaplan-Meier survival analysis for MACE in high or low TMAO subgroups.

The primary endpoint of long-term CV death occurred in 13 (5%) out of 292 patients, for whom the TMAO levels were available. Eleven of these patients who died had high TMAO values and 2 patients had low TMAO values (Table 4). Higher plasma TMAO level was associated with a crude 21.3-fold increased CV-related mortality risk (HR = 21.33, 95%CI: 4.71–96.48; *p* < 0.001; Table 5). For following adjustments, we used a multivariate Cox regression model to identify independent variables for long-term CV death (Table 6). Patients with high TMAO levels had 11.6-fold higher risk to experience CV death within seven yr as compared to those with lower TMAO values in the multivariate Cox regression model (adjusted HR = 11.62, 95% CI: 2.26–59.67; *p* = 0.003; Table 6). Kaplan-Meier survival analysis showed a significant separation of curves between patients with high and low TMAO levels (*p* < 0.001) (Figure 4A). The highest risk to die was found in a subgroup of patients with DM and high TMAO levels (DM high vs. low TMAO: 27.3 vs. 2.6%, *p* = 0.004; none DM: 11.1 vs. 0.5%; *p* < 0.001; Figure 4B). Additionally, Kaplan-Meier survival analysis showed a significant separation of curves between patients with high and low TMAO levels for MACE (*p* < 0.001) (Figure 4C).

**Table 4:** Event data.

**Event data n (%)**	**Overall** ***N*** **= 292 (100)**	**Low TMAO** ***N*** **= 225 (77)**	**High TMAO** ***N*** **= 67 (23)**	***p*** **-value**
MACE (1 year)	23 (8)	18 (8)	5 (7.5)	0.886
Myocardial infarction (1 year)	6 (2)	4 (1.8)	2 (3.0)	0.538
Cardiovascular death (1 year)	1 (0.3)	0 (0)	1 (1.5)	0.067
Long-term cardiovascular death (7 years)	13 (4.5)	2 (0.9)	11 (16.4)	**<0.001**

*Data are reported as n (number of patients) and percentages; Chi Square test used for the comparison. P value < 0.05 is presented bold. MACE, Major cardiac adverse events*.

**Table 5:** Univariate Cox regression for prediction of long-term cardiovascular mortality.

**Variable**	**HR**	**95%CI**		***p*-value**
High TMAO (μmol/L)	21.33	4.71	96.48	**<0.001**
Gender (male)	1.587	0.448	5.161	0.442
Previous MI	3.085	1.036	9.186	**0.043**
Age	1.077	1.028	1.127	**0.002**
Diabetes	4.190	1.402	12.523	**0.010**
Peripheral vascular disease	3.273	0.724	14.788	0.123
Prior PCI	0.042	0.000	154.219	0.450
Smoking	0.443	0.148	1.320	0.144
Platelets	1.000	0.992	1.007	0.919
Hemoglobin (g/dL)	0.685	0.542	0.865	**0.001**
Creatinine levels in admission (mg/dl)	2.229	1.359	3.656	**0.002**
Beta-blocker (BB)	0.407	0.089	1.853	0.245
Antidiabetics	2.005	0.551	7.292	0.291
Calcium channel blockers (CCB)	2.216	0.490	10.024	0.302

*P value < 0.05 is presented bold. HR, hazard ratio; CI, confidence interval*.

**Table 6:** Multivariate Cox regression model for prediction of long-term cardiovascular mortality.

**Variable**	**HR**	**95%CI**		***p*** **-value**
High TMAO (μmol/L)	11.615	2.261	59.666	**0.003**
Gender (male)	0.919	0.223	3.783	0.907
Age > 65 years	1.041	0.982	1.103	0.176
Previous MI	2.365	0.662	8.452	0.185
Diabetes	2.306	0.709	7.508	0.165
Hemoglobin (g/dL)	0.934	0.704	1.240	0.638
Creatinine levels in admission (mg/dl)	1.122	0.579	2.173	0.733

*P value < 0.05 is presented bold. HR, hazard ratio; CI, confidence interval*.

## Discussion

The present study was conducted to evaluate the association of and TMAO with CV-related and all-cause mortality in patients with ACS. It demonstrates that high concentration of TMAO in the plasma at the time of ACS is associated with 12-fold increased long-term CV mortality. Additionally, high concentration of TMAO in patients with DM is associated with a very high, 27% incidence of seven-yr CV mortality.

TMAO has recently been extensively investigated for its prognostic value. High levels of plasma TMAO were found to be associated with poor prognostic outcomes in several conditions, including CAD, HF, PAD, COPD, CKD and psoriasis (32–38). Moreover, some previous reports have also shown the association between TMAO and CV events in patients with ACS (39, 40). A number of studies and meta-analyses confirmed the association between higher concentration of TMAO and higher risk of MACE, including CV and all-cause death and mortality during follow-up periods of up to 4 years (14, 21, 41–46). Interestingly, higher levels of TMAO were related to increased incidence of all-cause mortality in a dose-dependent manner, namely 7.6% per each 10 μmol/L increment of TMAO in a median follow-up period of 4.3 yr (47). In our study, focusing on a very homogenous population of ACS patients, elevated levels of TMAO (≥4 μmol/L) were associated with a 12-times higher risk of CV death during the longest to our knowledge follow-up time of seven yr. Of interest, our study also provides novel evidence that high levels of TMAO were detected more frequently in patients with a history of MI or previous PCI as well as with post-PCI revascularization. Another important finding in our study is the strong association between DM and high levels of TMAO, which is in line with other studies (14, 48, 49). Recently, it has been demonstrated that circulating TMAO levels were independently associated with age, BMI, and diabetes status in animal model (50). It should be pointed out that although our patients had an optimal medical treatment at discharge (statins, BB, ACE-I, potent platelet inhibition), the residual risk with high TMAO is an independent predictor.

Our study shows that TMAO cut-off of 4 μmol/L was associated with a very high (99%) negative predictive value, 85% sensitivity, 80% specificity and an acceptable c-index of 0.78, underlying the effectiveness of TMAO as a predictive tool. Further studies are now necessary to investigate whether specific treatments or lifestyle modifications according to this cut-off would improve patient outcome.

It is interesting to speculate, which underlying mechanisms might be responsible for the association between high plasma TMAO and increased CV death. There is an ongoing debate whether TMAO is a mediator or merely a marker of cardiovascular pathology. Experimental data on cardiovascular effects of TMAO provide contradictory results (51–54). TMAO has been suggested to promote development of atherosclerotic plaque, alternate macrophage and endothelial cell phenotype and promote platelet reactivity (52, 55, 56). TMAO has also been implicated in the regulation of glucose, sterol and lipid metabolism which are associated with CAD (57–59). Notably, some studies not only show no negative effects related to TMAO, but also positive effects are observed (60, 61). Namely, in animal model high TMAO is suggested to decelerate aortic lesion formation and therefore may play a protective role in atherosclerosis progression (53). Additionally, TMAO fed rats presented not only lower systolic and diastolic blood pressure, but also downward trend of plasma NT-proBNP and raised LVEF (62, 63).

In this study, we have found that higher levels of TMAO were associated with increased all-cause mortality. However, plasma TMAO estimates for diagnostic accuracy were low. We also demonstrated that higher creatinine levels were associated with increased levels of plasma TMAO, a finding also observed by others (64). Nevertheless, in the multivariate analysis, only TMAO levels were revealed as an independent predictor of CV mortality.

Accumulating evidence suggests that concomitant medication is associated with higher plasma levels of TMAO. A significant increase in TMAO levels was observed among subjects using beta-blockers (BB), calcium channel blockers (CCB), statins and diuretics, but not metformin in one study (59). Indeed, patients with high TMAO levels in our study were also administered more frequently CCB and antidiabetic drugs, which might only specify DM disease associated treatment. In contrast to the published observations, BB use corresponded with low rather than high levels of TMAO in our study. Summing up, it is unclear whether specific or concomitant treatment can modulate TMAO levels. Therefore, further studies are needed to clarify this issue.

Building on the results of the present study we postulate that TMAO is a more accurate biomarker of CV pathology. It should be also underlined that most clinical studies investigating that issue have been carried out in patients with known CVD and in high-risk patients. Still, no standardized normal level and life-trend of TMAO has been studied in the general population and no prospective cohort study has been carried out in patients at different levels of cardiovascular risk.

## Study Limitations

The major limitation is the observational design of the study. Despite efforts to adjust for baseline differences using multivariate Cox regression analysis, a potential bias cannot be ruled out. Moreover, dietary consumption of TMAO precursors such as choline, betaine, L-carnitine may be influenced by sex, age, health condition or glycemic status. As we did not have the data of the daily diets of the patients, we also cannot exclude the potential influence of the used medications during hospitalization and diets by studied subjects. The paper does not distinguish between target and not target vessel revascularization due to the limitations of the database. Additionally, plasma sampling shortly after trauma or any other intervention may influence the concentration of TMAO (49, 59).

## Conclusion

In the current study, we demonstrated that in patients experiencing ACS, who were referred to PCI, the concentration of TMAO is independently associated with long-term CV mortality: patients with high TMAO levels (>4 μmol/L) were at 11-times higher risk to experience CV-related death than those with low TMAO. Furthermore, TMAO with a similar accuracy predicted all-cause death. Additionally, individuals suffering from DM had a higher concentration of TMAO, which was independently associated with increased risk of CV mortality. Future studies are necessary to delineate whether TMAO represents a pure marker of disease severity or a modifiable risk factor.

## Data Availability Statement

The raw data supporting the conclusions of this article will be made available by the authors, without undue reservation.

## Ethics Statement

The studies involving human participants were reviewed and approved by the Ethics Committee of the Medical University of Vienna approved the study protocol in accordance with the Declaration of Helsinki. The patients/participants provided their written informed consent to participate in this study.

## Author Contributions

MU evaluation of TMAO plasma concentrations. CE, JJ-P, DJ, AG, MW, MU, AT, IL, JS-M, and MP writing—original draft preparation. CE, DJ, MU, JS-M, and MP writing—review and editing. CE visualization. MU, JS-M, and MP supervision. All authors contributed to the article and approved the submitted version.

## Funding

This work was implemented with CEPT infrastructure financed by the European Union-the European Regional Development Fund within the Operational Program Innovative economy for 2007–2013.

## Conflict of Interest

The authors declare that the research was conducted in the absence of any commercial or financial relationships that could be construed as a potential conflict of interest.

## Publisher's Note

All claims expressed in this article are solely those of the authors and do not necessarily represent those of their affiliated organizations, or those of the publisher, the editors and the reviewers. Any product that may be evaluated in this article, or claim that may be made by its manufacturer, is not guaranteed or endorsed by the publisher.
